# Importance of proper diagnosis for management: multifocal choroiditis mimicking ocular histoplasmosis syndrome

**DOI:** 10.1007/s12348-010-0016-4

**Published:** 2011-03-16

**Authors:** Elham Hatef, Peykan Turkcuoglu, Mohamed Ibrahim, Yasir Sepah, Matthew Shulman, Jangwon Heo, Jeong Hee Lee, Roomasa Channa, Afsheen Khwaja, Zubir Rentiya, Syed Mahmood Shah, Diana V. Do, Quan Dong Nguyen

**Affiliations:** 1 Wilmer Eye Institute, Johns Hopkins University School of Medicine, 600 North Wolfe Street–Maumenee 745, Baltimore, MD 21287 USA; 2 Department of Ophthalmology, Inonu University School of Medicine, Malatya, Turkey; 3 Flaum Eye Institute, University of Rochester School of Medicine, Rochester, New York, USA

**Keywords:** Multifocal choroiditis, Ocular histoplasmosis syndrome

## Abstract

**Purpose:**

The study aims to evaluate a series of patients with initial diagnosis of ocular histoplasmosis syndrome (OHS) with progression and response to treatments consistent with multifocal choroiditis (MFC).

**Methods:**

Retrospective review of nine patients referred for management of recurrent OHS lesions. Serology panel was conducted to rule out autoimmune and infectious causes.

**Results:**

Clinical examination revealed multiple small, punched-out peripheral chorioretinal scars, and peripapillary atrophy. Histoplasma antigen/antibody was negative in all patients. Fluorescein angiography and optical coherence tomography confirmed active inflammation in five patients. Immunomodulatory therapy (IMT) was initiated to control active inflammation. While on IMT, visual acuity stabilized or improved in three patients with no recurrence of CNV or lesion activities over the follow-up period.

**Conclusions:**

MFC may initially masquerade as OHS. Clinical characteristics of recurrent MFC and absence of histoplasma titer may lead to consideration of IMT and other proper treatments for MFC.

## Introduction

Differentiating between multifocal choroiditis (MFC) and ocular histoplasmosis syndrome (OHS) may be challenging, especially if based solely on clinical examination. Both may present with chorioretinal (CR) lesions and lack of anterior chamber (AC)/vitreous inflammation [1]. Punched-out chorioretinal scars, peripapillary scarring, and choroidal neovascularization are present in both conditions [2]. However, as the management of OHS differs tremendously from that of MFC, it is important to make proper diagnosis. The index study evaluates a series of patients whose initial diagnosis was OHS. Patients underwent extensive review of systems, uveitis diagnostic survey, a comprehensive examination, and targeted evaluation. The subsequent progression and response to treatments in all patients were consistent with MFC.

## Materials and methods

Nine patients were referred from January 2008 to June 2010 to one of the authors (QDN) for management of OHS. Patients were diagnosed and managed as subjects with OHS in their primary institutes; laser photocoagulation/vascular endothelial growth factor (VEGF) antagonists for recurrent choroidal neovascularization (CNV) and episodic prednisone for lesions causing visual disturbances were prescribed. At the Wilmer Eye Institute, serology panel was conducted to rule out autoimmune and infectious causes. Fluorescein angiography (FA) and optical coherence tomography (OCT) were performed at initial and subsequent visits.

The charts of eligible patients were reviewed to include data regarding demographic features of each patient, previous diagnosis of OHS and any concomitant diagnosis, history of any relevant treatment to disease, and the length of follow up. The results of comprehensive ocular examination, serologic workup for all possible inflammatory and infectious conditions, as well as FA and OCT were reviewed. Final diagnosis and treatment applied for each patient was documented as well. Statistical summaries for baseline characteristics were reported. The final diagnosis, treatment regimen, and final outcomes for each patient were presented.

## Results

Eight women and one man with an age range of 26–69 years (median: 38 years) were included in the study. Six patients had a previous diagnosis of OHS, two had a likely diagnosis of OHS or punctuate inner choroidopathy (PIC), and one had a questionable diagnosis of uveitis and an inflammatory/infectious process such as sarcoidosis or OHS. Seven of the patients had a history of bevacizumab injection to control CNV, one of whom also received laser photocoagulation and photodynamic therapy; one had photodynamic therapy as well as intraocular injection of triamcinolone acetonide; the other one had photodynamic therapy prior to bevacizumab injection. One patient had a history of only laser photocoagulation to control CNV. The patients were followed up at our institute for a period of 1.18–14.51 months (median; 3.16 months).

**Fig. 1:**
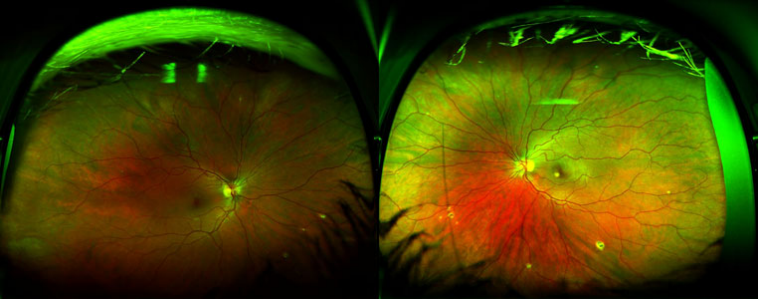
The Optos™ P-200° digital fundus image of patient 6 illustrates chorioretinal lesions inferiorly in both eyes. Lesions in the left eye are more prominent than those in the right eye. There is a yellowish lesion just inferonasal to foveal center of the left eye

**Fig. 2:**
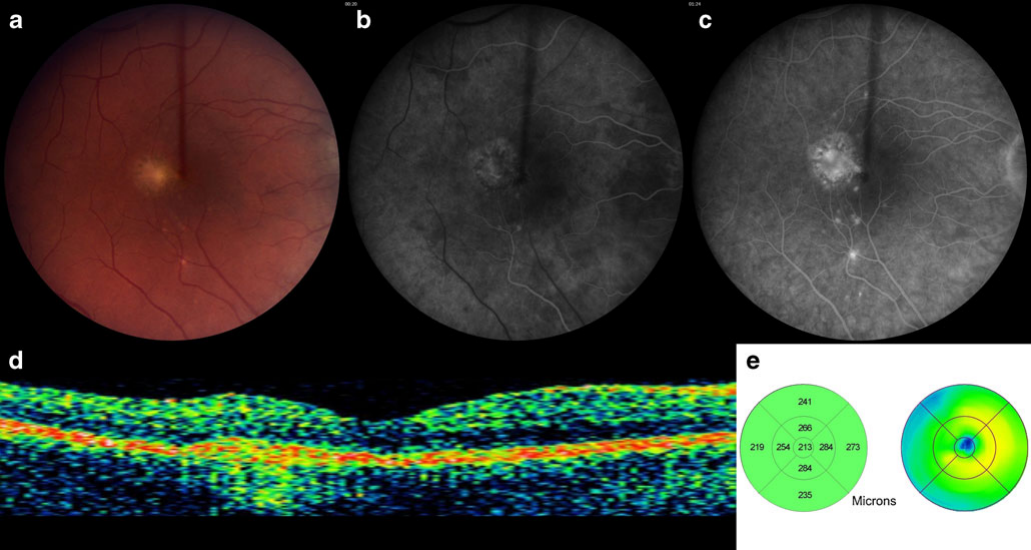
**a** Fundus image of the right eye of patient 5 illustrates multiple chorioretinal lesions, which concentrates within the macula. There is a deep chorioretinal scar on the temporal aspect of foveal center with no subretinal fluid. **b**, **c** Fluorescein angiography of right eye demonstrates classical choroidal neovascularization (CNV) filling pattern with chorioretinal anastomosis in the center without prominent leakage in early **b** and mid **c** phases. **d**, **e** Optical Coherence Tomography of right eye shows hyperreflective substance within the retinal pigment epithelium (RPE)/choroid complex, with no intra-retinal edema

We carried out a comprehensive ocular examination as well as serologic workup for all possible inflammatory and infectious conditions based on the clinical findings. Multiple small, punched-out peripheral CR scars and peripapillary atrophy were noted in all patients. No AC/vitreous inflammation was detected. None of them had active CNV at the time of initial visit (Figs. 1 and 2). Histoplasma antigen/antibody for all subjects was performed in the same clinical laboratory through the Johns Hopkins University and Hospitals, and both antigen and antibody were negative in all patients.

Rheumatoid panel and serology for *Herpes simplex* as well as *Herpes zoster* were also negative. FA and OCT confirmed active inflammation (choroiditis without CNV) in five patients. Table 1 shows the clinical and imaging findings as well as serologic evaluation for each patient.

**Table 1:** Demographic, clinical, and serologic characteristics of the patients

Case no./age, yr/race/sex/affected eye	Visual acuity		F/U (mo)	AC/vitreous reaction at initial visit	Funduscopic findings at initial visit	Serologic findings at initial visit	Imaging findings at initial visit	Treatment and clinical course
	Initial	Final						
1/47.57/W/F/BE	20/20 RE 20/400 LE	20/16 RE 20/250 LE	14.51	No evidence of iridocyclitis; no inflammatory cells in the vitreous	BE: Peripapillary atrophy; multiple pigmented, chorioretinal lesions scattering in the peripheral retina	Histoplasma Antigen: N	**FA:** RE: No evidence of any leakage within the foveal area; **leakage of two isolated lesions**	**Past Ocular Treatment**
					**RE: 2 small yellowish, deep chorioretinal lesions, 1 superonasal to the foveal center, 1 inferotemporal to the foveal center**	Histoplasma Antibody: N	LE: staining of the fibrotic scar, no active leakage	Laser photocoagulation and photodynamic therapy for CNV as well as intravitreal injection of bevacizumab
					LE: A large fibrotic scar in macula	Muramidase (Lysozyme): N	**OCT:** RE; **RPE disruption in areas corresponded to the two lesions superonasal and inferotemporal to the fovea**	**Treatments at the Wilmer Eye Institute**
						ACE: N		Methylprednisolone 1,000 mg/IV a day for 3 days. Prednisone 60 mg/PO a day tapered to 12.5 mg/daily over the period of 6 months; mycophenolate mofetil 2,000 mg/PO daily increased to 2,500 mg/PO daily after 4 months. Despite an increase in the dose of mycophenolate mofetil, on repeated OCTs, there were enlargements of the 2 lesions in the right eye compared to previous OCTs
						RPR Screen: NR		The patient was then enrolled in a clinical trial of local treatment of sirolimus, which helped to reduce the size of the lesions and allowed tapering of prednisone
						FTA-ABS-Serum: NR		
						ANA Screen: N		
						Anti-DNA: N		
						RNP Antibody: N		
						Smith Antibody: N		
						Anti-RO (SS-A): N		
						Anti-LA (SS-B): N		
						SCL-70: N		
						EBV IgG: P		
						Lyme Disease Antibody: N		
2/31.33/W/F/BE	20/80 RE 20/63 LE	20/40 RE 20/32 LE	3.16	No evidence of iridocyclitis; no inflammatory cells in the vitreous	**RE;** Several chorioretinal lesions within and outside of arcade, nasal to optic nerves and in the inferonasal quadrants	Histoplasma Antigen: N Histoplasma Antibody: N	**FA:** No leakage.	**Past Ocular Treatment**
					LE; Few chorioretinal scars within and outside of arcades, a curvilinear area of chorioretinal lesions in temporal area consistent with MFC	HSV type I IgG: P	**Autofluorescence**: **areas of hyperfluorescence surrounding various lesions in RE**	Prednisone 80 mg/daily for RE involvement. Methotrexate was added for 3 months after LE involvement. Methotrexate was discontinued. With a strong suspicion of PIC she was kept on prednisone 80 mg/daily
						HSV type II IgG: N	**OCT:** RE; Hyper reflective scar tissue involves the foveal center, starting from sub-RPE level extending to the inner plexiform layer	**Treatment at the Wilmer Eye Institute** Mycophenolate mofetil 1 g/twice a day. Prednisone tapered after 10 days of administration of mycophenolate mofetil. Three months later, prednisone tapered to 20 mg. During the last visit, funduscopic examination revealed stable findings of multiple chorioretinal lesions mainly in the posterior pole. There had been also a decrease in the amount of hyperfluorescence on autofluorescence, and a decrease in the amount of RPE disruption on OCTs in both eyes
						Muramidase (Lysozyme): N		
						ACE: N		
						RPR Screen: NR		
						FTA-ABS-Serum: NR		
						VZV IgG Antibody: P		
						Toxoplasma IgM: N		
						Toxoplasma IgG: N		
						EBV IgM: N		
3/50.14/W/F/BE	20/250 RE 20/25 LE	20/160 RE 20/25 LE	1.18	No evidence of iridocyclitis; no inflammatory cells in the vitreous	BE: Multiple hypopigmented chorioretinal spots in the mid periphery **RE: Edema and subretinal fibrosis through the fovea**	Histoplasma Antigen: N Histoplasma Antibody: N	**FA**: RE: **Hyperfluorescence and leakage from many hypopigmented spots in the mid periphery as well as mild leakage from some of the vasculature in the peripheral retina**; no optic disk leakage or staining, hyperfluorescence in the fovea corresponding to the area of fibrosis	**Past Ocular Treatment**
					LE: Pigmentary changes without edema through the fovea	ANA Screen: N	**LE: Leakage from the hypopigmented peripheral spots**; no optic disk leakage or staining, no macular edema	Bevacizumab 4 injections for CNV.
						Anti-DNA: N	**OCT:** RE: **Intraretinal edema through the center of the fovea**	**Treatment at the Wilmer Eye Institute**
						RNP Antibody: N	LE: very mild intraretinal edema	We recommended a consult with her hematologist/oncologist to evaluate for a possible hematologic underlying disease prior to initiation of potential treatment for multifocal choroiditis
						Smith Antibody: N		
						Anti-RO (SS-A): N		
						Anti-LA (SS-B): N		
						HSV type I IgG: N		
						HSV type II IgG: N		
						VZV IgG Antibody: N		
						Toxoplasma IgM: N		
						Toxoplasma IgG: N		
						EBV IgG: N		
4/26.91/W/F/BE	20/200 RE 20/20 LE	20/125 RE 20/20 LE	1.68	No evidence of Iridocyclitis; no inflammatory cells in the vitreous	RE: Multiple small discrete pigmented chorioretinal lesions scattered in posterior pole and throughout the peripheral retina. Atrophic changes and subretinal fibrosis in the macula	Histoplasma Antigen: N Histoplasma Antibody: N	**FA:** RE: Multiple small lesions scattered in posterior pole and throughout the peripheral retina without leakage. Scar staining noticed at the foveal center	**Past Ocular Treatment**
					LE: Few scattered chorioretinal lesions limited mainly to nasal area of optic nerve. Ring of pigment next to the foveal center with no subretinal fluid or hemorrhage	Muramidase (Lysozyme): N	LE: Classic CNV filling pattern noticed without leakage at late phase	Bevacizumab2 injections for CNV
						ACE: N	**OCT:** RE: Multiple lesions involve different layers of retina and sub-RPE in some areas, including foveal center which result in intra retinal edema, more pronounced nasally	**Treatment at the Wilmer Eye Institute**
						RPR Screen: NR	LE: presence of a PED	The patient was monitored. Immunomodulatory therapy would be started to decrease the risk of inflammations in case of recurrent CNV in LE. Vascular endothelial growth factor antagonist would be employed as well when there is recurrent CNV
						FTA-ABS-Serum: NR		
						ANA Screen: N		
						HSV type I IgG: N		
						HSV type II IgG: N		
						EBV IgG: P		
						EBV IgM: N		
						Toxoplasma IgM: N		
						Toxoplasma IgG: N		
5/38.01/W/F/BE	20/63 RE 20/20 LE	20/25 RE 20/16 LE	2.83	No evidence of iridocyclitis; no definite inflammatory cells in the vitreous	RE: Multiple chorioretinal lesions, much more in the nasal aspect of retina. Deep chorioretinal scar on the temporal aspect of foveal center with no subretinal fluid and two lesions in the temporal peripheral retina	Histoplasma Antigen: N Histoplasma Antibody: N	**FA:** RE: chorioretinal anastomosis in foveal center without significant leakage	**Past Ocular Treatment**
					LE: Chorioretinal lesions in the nasal aspect of the retina. Macula appeared normal		**OCT:** RE: Scar tissue involved RPE and photoreceptor layer, no intraretinal edema	Photodynamic therapy and intraocular corticosteroid followed by one intravitreal injection of bevacizumab
							**AF:** RE: **hyperfluorescence surrounding several chorioretinal lesions**	**Treatment at the Wilmer Eye Institute**
								Mycophenolate mofetil 1 g twice a day. During the most recent visit, the lesions appeared to be much less active. On FA, there was no evidence of leakage in the macula of either eye
								Also, there had been much decrease in the amount of fluorescence of macular lesions in RE on AF
6/31.96/W/F/BE	20/20 RE 20/80 LE	20/125 RE 20/125 LE	5.69	No evidence of Iridocyclitis; no inflammatory cells in the vitreous	**BE: Chorioretinal lesions inferiorly, LE worse than RE**	Histoplasma Antigen: N		**Past Ocular Treatment**
					**LE: yellowish lesion inferonasal to foveal center**	Histoplasma Antibody: N		Bevacizumab injections for CNV
								**Treatment at the Wilmer Eye Institute**
								Prednisone 60 mg/daily for 1 month tapered by 10 mg/daily every 3 weeks. Mycophenolate mofetil 1,000 mg twice a day and increased to 2,500 mg daily after 2 months; cyclosporine (4 mg/kg) was also started. During the most recent visit, AF did not provide any new lesion
7/36.16/W/F/BE	20/200 RE 20/25 LE	20/200 RE 20/25 LE	5.23	No evidence of iridocyclitis	RE: Disciform scar in foveal center and curvilinear distribution of multiple chorioretinal lesions in peripheral retina, especially nasally	Histoplasma Antigen: N Histoplasma Antibody: N	**FA:** There was no leakage on the FA to suggest active CNV	**Past Ocular Treatment**
				no definite inflammatory cells in the vitreous	LE: curvilinear distribution of multiple chorioretinal lesions in peripheral retina especially nasal aspect	RPR Screen: NR	**OCT:** RE: Disciform scar in foveal center involved RPE and photoreceptor layer, no intra retinal edema	Photodynamic therapy (3 times) and 4 intravitreal injections of bevacizumab in RE. 1 intra vitreal injection of bevacizumab in LE
						FTA-ABS-Serum: NR	**LE: 5 lesions surrounding foveal center with involvement of RPE and disruption of the photoreceptor layer**	**Treatment at the Wilmer Eye Institute**
						ANA Screen: P		Intravenous methyprednisolone daily for 3 days. Prednisone 60 mg/daily for 2 weeks then tapered to 9 mg/daily in a period of 4 months
					**There were 5 lesions with blurry borders surrounding the foveal center**	Anti-DNA: N		
						RNP Antibody: N		
						Smith Antibody: N		
						Anti-RO (SS-A): N		
						Anti-LA (SS-B): N		
						Lyme Disease Antibody (ELISA): equivocal for antibody		
						Lyme Disease Antibody (Western Blot): N		Mycophenolate mofetil 1,000 mg twice a day for 3 months, then increased to 1, 500 mg twice a day. During the last visit, on AF, there appeared to be decreased hyperfluorescence surrounding the lesion in RE, especially in nasal aspect of the retina. AF inLE appeared to be stable. There was no clear leakage seen on the macula of either eye.
8/69.22/W/F/BE	20/200 RE 20/400 LE	20/200 RE 20/250 LE	2.30	No evidence of iridocyclitis; no inflammatory cells in the vitreous	BE: Peripapillary atrophy, fibrotic scars in macula and multiple chorioretinal lesions in the peripheral retina; no subretinal fluid or heme	Histoplasma Antigen: N Histoplasma Antibody: N	**FA:** There was no leakage on the FA to suggest active CNV	**Past Ocular Treatment**
						Muramidase (Lysozyme): N	**OCT:** BE: Scars in foveal center involved RPE and photoreceptor layer; no intra retinal edema	5–6 intravitreal injections of bevacizumab in LE
						ACE: N		**Treatment at the Wilmer Eye Institute**
						RPR Screen: NR		
						FTA-ABS-Serum: NR		Monitoring the patient and evaluations of tuberculosis. No IMT was initiated
9/61.97/W/M/LE	20/20 RE 20/400 LE	20/20 RE 20/320 LE	12.20	No evidence of iridocyclitis; no inflammatory cells in the vitreous	LE: Pigmentary changes as well as laser scar and atrophic changes within macula	Histoplasma Antigen: N Histoplasma Antibody: N	**FA:** There was no leakage on FA to suggest active CNV	**Past Ocular Treatment**
							**OCT:** BE: Scar in foveal center involved RPE and photoreceptor layer; no intraretinal edema	Laser photocoagulation for CNV in LE
								**Treatment at the Wilmer Eye Institute**
								**T**he patient was monitored as there was no evidence of active inflammation

Bold text in the table represents clinical findings that were suggestive of active inflammation

*F/U* follow-up, *AC* anterior chamber, *W* white, *F* female, *M* male, *RE* right eye, *LE* left eye, *BE* both eyes, *Mo* month, *P* positive, *N* negative, *NR* nonreactive, *ANA* antinuclear antibody, *HSV* Herpes simplex virus, *ACE* angiotensin-converting enzyme, *RPR* rapid plasma regain, *FTA-ABS* fluorescent treponemal antibody absorption, *VZV* Varicella zoster virus, *EBV* Epstein–Barr virus, *FA* fluorescein angiography, *AF* auto-fluorescein, *OCT* optical coherence tomography, *CNV* choroidal neovascularization, *IV* intravenous, *PO* per oral, *PED* pigment epithelial detachment, *IMT* immunomodulatory therapy

Based on the clinical examination and the result of serologic evaluations that were nonrevealing for other causes, a diagnosis of immune-mediated MFC was made in seven patients. Patient 8 had a history of positive PPD testing and ocular finding which might be consistent with tuberculous choroiditis. We recommended the patient to be evaluated for tuberculosis infection as well. For patient 9, based on the clinical findings and negative serology for histoplasmosis, it was most likely that he had CNV, which is idiopathic in nature. There did not seem to be any active CNV at the time of examination. Therefore, the patient was advised to monitor his vision closely and be re-examined regularly to look for any signs of CNV activity.

Immunomodulatory therapy (IMT) was initiated to control the inflammation in five of the eight patients (patients 1, 2, 5, 6, and 7). They received treatment from 1.84 to 6.22 months. Patient 3 had a complicated medical history of scleroderma, graft-versus-host disease after an allogenic bone marrow transplant for leukemia, history of cytomegalovirus encephalopathy, and shingles. She was on mycophenolate mofetil for scleroderma and graft-versus-host disease at the time of initial visit. Considering the underlying hematologic disease, we recommended a consultation with her hematologist/oncologist prior to starting treatment for MFC. For patient 4, based on examination and ancillary testing, it was most likely that she had primary MFC rather than OHS or toxoplasmosis. We decided to monitor the patient and would begin IMT treatment to decrease the risk of recurrent inflammation if active choroiditis or recurrent CNV reappeared. In addition, VEGF antagonist would be employed as needed to provide rapid control of any recurrent CNV.

While on IMT, visual acuity stabilized or improved in patients 2, 5, and 6 with no recurrence of CNV or lesion activities over the follow-up period. These three patients were followed up for a period of 3.16, 1.84, and 5.69 months, respectively. Patient 1 had an episode of visual disturbance in the right eye which was accompanied with an enlargement of two previously detected lesions on OCT. The disease activation was not controlled by increase in the dose of IMT; therefore, the patient was enrolled in a clinical trial of local treatment of another IMT. Patient 7 had a worsening of visual acuity in the right eye from 20/200 to 20/320 after initiation of IMT (mycophenolate mofetil). Considering the short period of IMT treatment and slow treatment response in the case of IMT, we decided to continue our recommended treatment and follow up the patient every 4 weeks. The visual acuity improved to 20/200, 3 months after initiation of IMT and the dosage was increased to 1.5 g twice a day at the last visit. Patient 4 and 9 had stable disease at the most recent visits and were thus being observed on no IMT. As mentioned, patient 8 was being evaluated by the Infectious Diseases Service for possible tuberculosis infection. Since she did not have any active lesion, we decided to observe her until a definitive diagnosis was identified.

At the last visit, the visual acuity among all patients has changed from 20/20–20/250 (median; 20/80) to 20/16–20/200 (median; 20/125) in the right eye and from 20/20–20/400 (median; 20/63) to 20/16–8/320 (median; 20/32) in the left eye.

Additional details on IMT and treatment response for each patient are presented in Table 1. In addition, imaging studies of patients with diagnosis of MFC showed decrease in the amount of fluorescence of the lesions on autofluorescence among those being treated with IMT.

## Discussion

Patients with MFC may masquerade as OHS. The index study suggests that the diagnosis of OHS should not be considered *exclusively* unless accompanied by positive serology, absence of inflammation, and appropriate clinical course for OHS. Even then, the patients should be followed closely for progression of the lesions as the disease may be of different entities. Our patients had many features that resembled OHS. Peripheral and posterior pole scars like those seen in OHS were present in our patients. However, the chorioretinal lesions resulted from inflammatory processes may be similar to those in OHS, with pigmented borders and centers.

An enlarged blind spot at the time of presentation with acute lesions or thereafter when the lesions are inactive has been reported in patients with MFC [3]. For patient 5, the Amsler visual testing revealed the presence of an old scotoma in the nasal aspect of the field as well as a newer small scotoma in the temporal aspect of the right visual field. Multifocal chorioretinal lesions were seen in every patient. The lesions were generally smaller and more numerous than the typical histoplasma spots in OHS [4, 5]. There was a predominance of women similar to other reports on MFC [1, 2]. The histoplasma antigen/antibody, performed in the same laboratory was negative in all our patients. Previously, serologic testing for *Histoplasma capsulatum* used to have relatively low sensitivity with positive result in 15% of the patients [2]. The recently available serologic testing for *H. capsulatum* antigen is highly sensitive. If it is disseminated histoplasmosis in an immunocompetent patient, the sensitivities in serum and urine samples are 82% and 92%, respectively; specificity is 98% and its reproducibility is excellent [6]. In those with limited pulmonary disease, the sensitivities in serum and urine samples are 68.6% and 64.6%, respectively [7]. In antibody test against *H. capsulatum*, the positive immunodiffusion reactions involve one or more specific precipitin bands. Of these bands, the first to appear in active histoplasmosis is the ″M″ band, which is seen in approximately 70% of proven cases. The M band is also seen in a number of patients with past infections and in 20% of those with recent *Histoplasma s*kin testing. The ″H″ band is usually seen in active and progressive histoplasmosis and almost always exists in the presence of the ″M″ band, although it is found less often in approximately 10% of proven cases (Quest Diagnostics Nichols Institute, 14225 Newbrook Drive, Chantilly, VA 20153, and Immuno Mycologics, Inc (Immy) [8]).

Evidence of Epstein–Barr virus (EBV) infection was detected in one of our patients, which is consistent with Tiedeman’s report [9] about the possible relationship between MFC and recent EBV infection.

The lesions observed in patients in the index study were also different from those in PIC, which typically do not have inflammatory signs, cells, or vitreous inflammation, and often constitute a cluster of macular lesions [1]. PIC patients have chorioretinal scars mostly in the posterior pole and the clinical course is less likely complicated by recurrent inflammation. The visual prognosis is usually stable unless CNV develops. Unlike PIC, patients with MFC have chorioretinal scars mostly in the mid-peripheral retina and a course characterized by waxing and waning inflammation that results in poor visual prognosis [10].

The imaging findings in the index study supported the MFC lesions and were consistent with the lesions described as MFC in other studies [11, 12]. Retinal pigment epithelium (RPE) was altered on OCT images. The hyper-fluorescent leakage on FA images as well as hypo-fluorescence on autofluorescene was consistent with previous descriptions of MFC. These spots were associated with chorioretinal scars and atrophy or absence of RPE cells detected in the posterior pole of affected eyes [11, 12].

Once the patients were thought to have MFC with recurrent diseases, corticosteroids and IMT were initiated, which led to full control (no recurrences) of inflammation and preservation of vision secondary to absence of active inflammation [13]. IMT also allowed tapering of dosages of corticosteroids to avoid long-term complications.

The current study emphasizes the importance of considering the possibility of other diagnoses when the disease has atypical characteristics and behavior. Based on our experience, when the actual chorioretinal lesions are inflamed in the presence of an inactive CNV, the diagnosis of OHS might be in doubt and should be reconsidered, and different treatment approach might be required.

However, we also recognize the many limitations of our study. The index study is a retrospective review of a small number of patients. Many of the patients were followed for only a short period of time. Longer follow-up time in a large group of patients will provide more information regarding the challenges in the diagnosis and management of MFC masquerading as other conditions such as OHS and will also help to confirm that treatment with IMT decreases recurrences of diseases, as expected in MFC, and not in OHS. We also recognize that many patients might not have presented with appropriate information and history to aid the referring physicians to consider MFC as possible etiologies.

Differentiation between MFC and OHS can be quite challenging, especially when both conditions are quiescent and there has been no previous examination or documentation of the appearance of the fundus [1]. Serologic testing for *H. capsulatum*, which is recently available with high sensitivity, should be employed to aid in the management of such patients, as absence of histoplasma titer and clinical characteristics of recurrent MFC may be more supportive for a diagnosis of (inflammatory) MFC and may lead to consideration of IMT and other appropriate treatments for MFC.
